# Family quality of life in early intervention: systematic review and meta-analysis

**DOI:** 10.3389/fped.2026.1866502

**Published:** 2026-06-24

**Authors:** Sara Pérez-Granado, Rocío Palomo-Carrión, Elena Pinero-Pinto

**Affiliations:** 1 Department of Physiotherapy, University of Seville, Seville, Spain; 2 Department of Nursing, Physiotherapy and Occupational Therapy, Universidad de Castilla-La Mancha - Campus de Toledo, Toledo, Spain; 3 Biomedicine Institute of Seville (IBIS), Seville, Spain

**Keywords:** developmental disabilities, early intervention, family centered-intervention, family quality of life (FQOL), quality of service

## Abstract

**Background:**

Family Quality of Life (FQOL) in Early Intervention (EI) has become a key factor for the evaluation of its results, since, in early ages, the family plays a fundamental role. The main objective of this review was to examine the scientific evidence on FQOL of families with children who attend EI services.

**Methods:**

This systematic review was conducted in the Pubmed, Scopus, Web of Science and CINAHL databases. Two meta-analyses were carried out using Cronbach's *α* of the measurement instruments and the means and standard deviations of FQOL.

**Results:**

The search produced 7,249 records, of which 10 were included. The statistical result of Cronbach's *α* was high, and that of FQOL was medium-high.

**Conclusions:**

Families participating in EI programs tend to report medium-to-high levels of FQOL. The factors that contribute to improving FQOL include: the supports provided by the professional, identification of strengths, satisfaction with the service, and transdisciplinary interventions focused on the family.

## 1 Introduction

Family Quality of Life (FQOL) can be defined as “a dynamic perception of family well-being, collectively and subjectively defined by its members, where the individual and family needs interact with each other” (1). The concept of FQOL is fundamentally explained by the contributions of ecological theory and systems theory, which are based on the interaction of the individual with their surroundings, the situations in which they participate directly, and their relationships with other individuals (1–3). This conception grants the family an essential role (3).

EI can be defined as a “set of interventions aimed at children aged 0 to 6, their families, and their communities, with the aim of addressing the temporary or permanent needs of children with developmental disorders or who are at risk of suffering from them” in which collaboration among children, families, and schools is crucial to effectively guide preschool development (4, 5). Developmental disorders can be defined as a group of conditions that begin during the developmental period. They are characterized by developmental deficits that produce limitations in personal, social, academic, or occupational areas of functioning (language, cognition, motor skills, or adaptive behavior) (6). EI in developmental disorders requires formal and informal support networks, considering the family system as a whole (7). EI appear to improve FQOL, especially in families of children with developmental delays (8). Family-informed EI improves parents' ability to respond to their child's needs, which reduces parental stress and increases satisfaction—positively impacting the family environment (9).

Data show that family quality of life not only improves as a child progresses, but also that a resilient, supported, resourceful, and less stressed family facilitates more effective early intervention (10). Therefore, FQOL and EI in children with developmental disorders appear to be closely related.

Although FQOL is increasingly recognized as important in EI, there is still no clear or consistent understanding of how it is defined, measured, or what factors influence it in families with children in EI. This limits the comparability and synthesis of findings across studies. This heterogeneity, particularly regarding the wide range of developmental conditions, functional levels, and service delivery contexts included under early intervention, complicates the interpretation of results. This heterogeneity further supports the need for a meta-analytic approach to synthesize available quantitative evidence (11). This gap is particularly relevant for families of children with developmental disorders, who often face unique and complex challenges during the early years of diagnosis and care (8, 12). Understanding how Early Intervention affects their quality of life is essential to improve clinical decision-making, guide service provision, and support family-centered practices (13, 14).

The objective of this systematic review and meta-analysis is to synthesize and analyze the scientific evidence regarding Family Quality of Life (FQOL) in families of children receiving Early Intervention (EI) services. Specifically, the systematic review aims to examine how FQOL is conceptualized in this context, identify the most commonly used assessment instruments, and describe the key factors reported in the literature that may influence family quality of life. In addition, the meta-analysis aims to quantitatively synthesize the available evidence on reported levels of FQOL in this population.

## 2 Methods

This study is a systematic review with meta-analysis examining Family Quality of Life (FQOL) in families of children receiving Early Intervention (EI) services. The review was registered in the International Prospective Register of Systematic Reviews (PROSPERO: CRD42024522283) and conducted following the Preferred Reporting Items for Systematic Reviews and Meta-Analyses (PRISMA) guidelines (15).

### 2.1 Eligibility criteria

The eligibility criteria were structured according to the PECO framework. The Population (P) included families with children with developmental disorders who attended EI (0–6 years). The Exposure (E) was participation in Early Intervention programs. No comparison group (C) was required. The Outcome (O) was Family Quality of Life (FQOL), assessed using validated instruments. Eligible study designs included observational studies (cross-sectional, cohort, and mixed-method studies) as well as pre–post intervention designs reporting quantitative FQOL data.

Exclusion Criteria

- Articles that were not published in English.
- Systematic reviews and academic studies (final projects leading to a degree).

### 2.2 Information sources and search strategy

A search was carried out in the Pubmed, Scopus, Web of Science and CINAHL databases. Predefined keywords related to developmental disorders, early intervention, and family quality of life were used. The complete search strategy for each database is provided in Supplementary Appendix S1.

### 2.3 Study selection

The study selection process was conducted independently by two authors (SPG, EPP), using Rayyan software. Any disagreement between these two researchers was solved by a third evaluator (RPC).

### 2.4 Data extraction

Data extraction was performed independently by two reviewers using a standardized data collection form. Any discrepancies were resolved through discussion, and no persistent disagreements required consultation with a third reviewer. The following information was extracted from each study: author and year of publication, study design, sample size, participant characteristics (including age, gender, and diagnosis), type of Early Intervention, measurement instruments used to assess Family Quality of Life (FQOL), and reported outcomes (means, standard deviations, and, when available, reliability indices such as Cronbach's *α*). Additional variables related to family context and intervention characteristics (such as support needs, satisfaction with services, and sociodemographic factors) were also collected when reported. Extracted data were summarized in tables to facilitate comparison across studies.

### 2.5 Evaluation of the methodological quality and risk of bias of the included studies

The methodological quality of the included studies was assessed using the Quality Assessment Tool for Observational Cohort and Cross-Sectional Studies (16) developed by The National Heart, Lung, and Blood Institute (NHLBI) (17). This tool examines potential sources of bias related to study design, including participant selection, measurement of exposure and outcomes, confounding variables, and statistical analysis. Each study was classified as having “good”, “fair”, or “poor” methodological quality based on predefined criteria. Two reviewers independently conducted the assessment, and discrepancies were resolved by consensus.

### 2.6 Quantitative data analysis

Two complementary meta-analyses were conducted. First, a meta-analysis of Cronbach's alpha coefficients was performed to assess the internal consistency of the FQOL measurement instruments across studies. This step ensured the reliability and comparability of the reported scores.

Second, a meta-analysis of standardized mean FQOL scores was conducted using reported means and standard deviations. When necessary, scale scores were normalized to a 1–5 range, with higher scores indicating better family quality of life.

Effect sizes and pooled estimates were calculated using random-effects models. Statistical heterogeneity was assessed using the Q statistic and the I^2^ index. Forest plots were generated to visualize individual and pooled effects.

All statistical analyses were performed using IBM SPSS Statistics version 28. A significance level of *p* < 0.05 and a 95% confidence interval were applied.

### 2.7 Reporting and data availability

Results are presented in accordance with PRISMA recommendations. Detailed search strategies and Supplementary Materials are available in the Supplementary Appendix S2.

## 3 Results

### 3.1 Study selection

**Figure 1:**
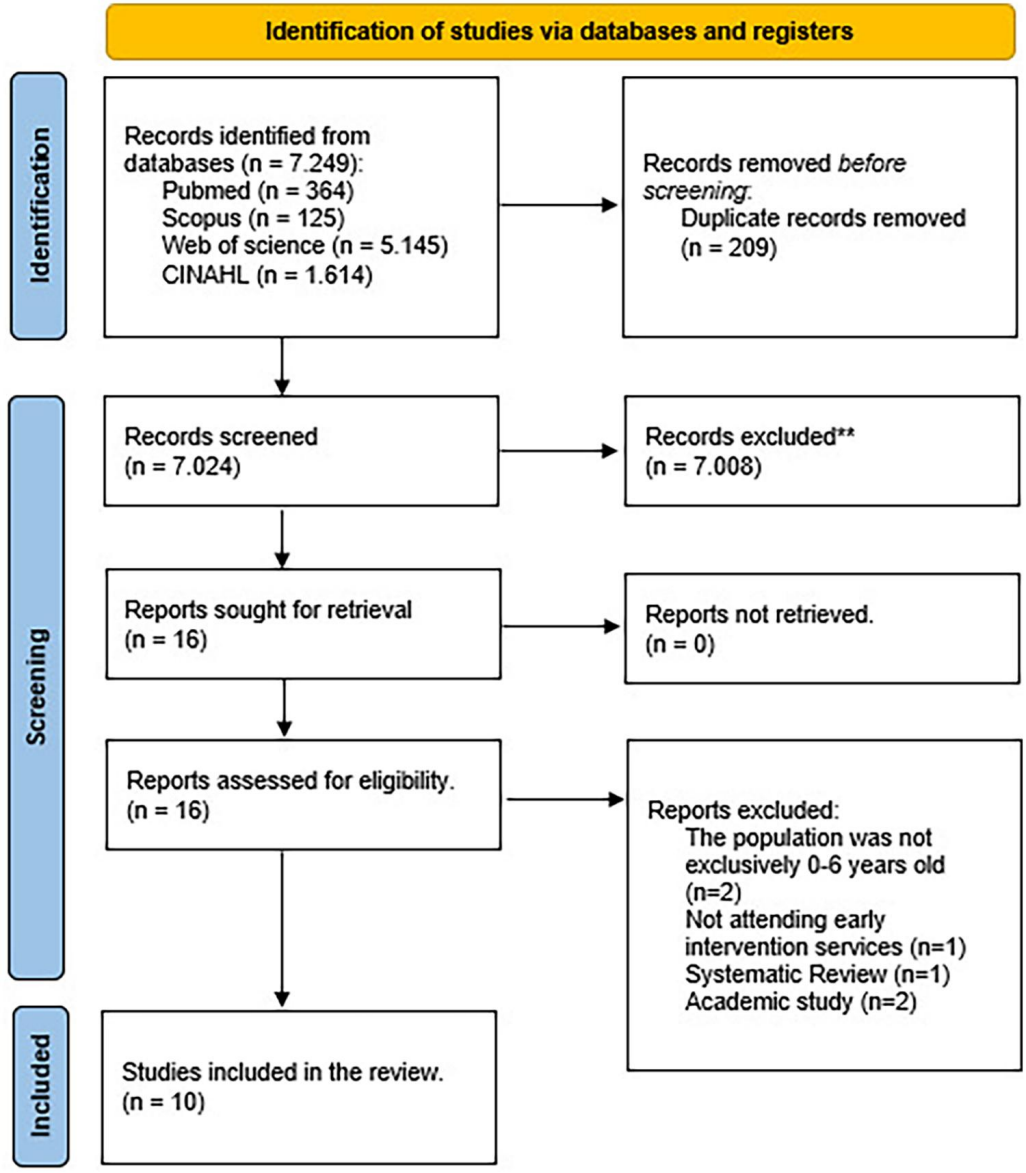
PRISMA flow chart (15).

The search produced a total of 7,249 records, of which 209 duplicates were discarded. After removing duplicates (*n* = 209) and screening titles and abstracts, 16 full-text articles were assessed, 10 studies met inclusion criteria and were included in the systematic review and meta-analysis (Figure 1).

### 3.2 Methodological quality and bias of the studies

The internal validity of these 10 articles was evaluated using the Quality Assessment Tool for Observational Cohort and Cross-Sectional Studies (16). To assess both the “cross-sectional and descriptive” articles and the “mixed methods” articles, Items 8 and 13 of the scale were classified as “not applicable”. For the longitudinal cohort study, all 14 items of the scale were applied. Thus, one article obtained the highest score (18) (8 out of a maximum of 12 points), one article obtained 7 out of 12 points (14), and seven articles obtained a score of 6 out of 12 points (7, 12, 13, 19–22). The longitudinal study obtained 10 out of 14 points (8) (Table 1).

**Table 1:** Results of the quality assessment tool for observational cohort and cross-sectional studies (16).

N°	Author and year	Obtained score	Methodological quality
1	Balcells-Balcells, A. et al. (13)	6/12	Medium
2	Epley, PH. et al. (14)	7/12	Medium
3	Wang, M. et al. (19)	6/12	Medium
4	Bhopti A. et al. (23)	6/12	Medium
5	Verger, S. et al. (7)	6/12	Medium
6	Chiu, SJ. et al. (8)	10/14	Good
7	García-Grau, P. et al. (12)	6/12	Medium
8	Mas, JM. Et al. (21)	6/12	Medium
9	Jemes-Campaña, IC. et al. (20)	6/12	Medium
10	Bagur, S. et al. (18)	8/12	Good

### 3.3 Characteristics of included studies

Considering the 10 articles included in the review, 6 of them used the *Beach Center Family Quality of Life Scale (BC-FQOLS)* (7, 8, 13, 14, 19, 22), one article employed the *Families in Early Intervention Quality of Life* (*FEIQoL)* (12), another article applied the *Family Quality of Life Scale for families with children under 18 years of age (CdVF-E)- Spanish or Catalan version* (21), and, lastly, two articles used the *Family Quality of Life Scale for families with children with Intellectual and/or Developmental Disabilities under 18 years of age (CdVF-ER)* (18, 20). The data extracted from each study are presented in Table 2.

**Table 2:** Descriptive data of population and selected studies.

N°	Author and year	*N* = parents	% female (children)	% age (children)	% diagnosis (children)	% relationship with the child	% age (parents)	Instruments	Cronbach's *α*	Type of study	FQOL Mean (SD)	Key findings
1	Balcells-Balcells et al. (13)	202	35.6	<3: 26.2	Intellectual disabilit	Mother: 7.8	<40: 63.9	BC-FQOLS-	0.96	Cross-sectional	3.66 (0.72)	EI satisfaction positively related to FQOL
				>3: 73.8	y: 42.1%	Father: 18.3	>40: 34.1					
						Other: 1.5						
2	Epley. et al. (14)	77	54.4	<3: 49.4	Developmental delay/Speech difficulty: 70.2	Mother: 96.1	NS	BC-FQOLS	0.94	Cross-sectional	4.40 (0.44)	Emotional well-being strongest predictor
				>3: 48.1		Father: 3.9						
3	Wang. et al. (19)	280	NS	<3: 66.8% | >4: 28.9%	Speech difficult: 49	NS	NS	BC-FQOLS	0.94	Cross-sectional	NS	Fathers: quality of life drops with severity; Mothers: rises with income.
4	Bhopti et al. (22)	72	NS	<4: 59.7	ASD: 26.4/Develompental delay: 15.3	Mother: 80.6	NS	BC-FQOLS	0.94	Mixed methods	4.02 (0.57)	Emotional well-being strongest predictor
				>4: 40.3		Father: 16.7						
						Others: 2.8						
5	Verger et al. (7)	166	NS	Mean ∼3.8	Speech difficulty/Develompental delay: 40	NS	Mean: 30.5–39.4	BC- FQOLS	0.88	Mixed methods	HG: 3.90/CG: 3.90	Different satisfaction profiles home group vs. center group
6	Chiu et al. (8)	142	25.8	<3: 39.4 >3: 60.6	Speech difficulty/Motor delay: 50%	Mother: 84.5	<40: 71.2	BC-FQOLS	0.93 T0. 0.96 T1-3	Cohort, prospective	3.59–3.95	FQOL increased until 6 months
						Father: 9.2	>40: 28.8					
						Other: 6.3						
7	García-Grau et al. (12)	250	27.37	<2: 25.2	Developmental/ motor delay: 40.4	NS	NS	FEIQoL	0.89	Cross-sectional	3.30 (0.52)	Lower scores in ASD families
				>2: 72.4								
8	Mas et al. (21)	280	31	<3: 28.2	Intellectual disability/developmental delay: 100	Mother: 77.2	M = 30. Range 22-58	CdVF-E	0.96	Cross-sectional	3.47 (0.46)	Income associated with FQOL
				>4: 65.1		Father: 20.3						
						Others: 2.5						
9	Jemes-Campaña et al. (20)	1,551	NS	NS	NS	NS	<40: 67.8	CdVF-ER	0.90	Cross-sectional	3.97 (0.62) *Data provided by the authors	Service quality predicts FQOL
							>40: 24.0					
10	Bagur et al. (18)	322	NS	<3: 36.9	Language delay: 38/ASD: 26.1	NS	<40: 58.3	CdVF-ER	0.91	Cross-sectional	3.91 (0.49)	Empowerment strongly related to FQOL
							>40: 41.5					
				>3: 63.1								

NS, Not Specified; FQOL, Family quality of life; BC-FQOLS, Beach Center Family Quality of Life Scale; EI, Early intervention; CdVF-ER, Family Quality of Life Scale for families with children with Intellectual and/or Developmental Disabilities under 18 years of age; FEIQoL, Families in Early Intervention Quality of Life.

In addition to FQOL, four articles measured the support needs of the families (8, 13, 14, 18), seven articles measured sociodemographic data (8, 12, 14, 18, 19, 21, 22), and three articles measured the satisfaction with the service (7, 20, 22).

### 3.4 Meta-analysis results

#### 3.4.1 Reliability (cronbach's *α*)

The pooled Cronbach's *α* was 0.932 (95% CI: 0.912–0.952), standard error (0.0102) (Table 3). The homogeneity test obtained a significance value of 0.086 (Table 4), whereas the *p* value was below 0.01 in the global Cronbach's test.

**Table 3:** Cronbach's α and FQOL estimations.

	Overall	Std. Error	*Z*	Sig. (2-tailed)	95% Confidence Interval		95% Prediction Interval	
					Lower	Upper	Lower	Upper
Cronbach's α	0.932	0.0102	91.215	0.000	0.912	0.952	0.880	0.983
FQOL	3.812	0.1776	21.462	0.000	3.464	4.160	3.392	4.232

a. Based on t-distribution.

**Table 4:** Cronbach's α and FQOL homogeneity test.

Overall	Chi-square (Q statistic)	df	Sig.
Cronbach's α	15,184	9	0.086
FQOL	3.808	8	0.874

**Figure 2:**
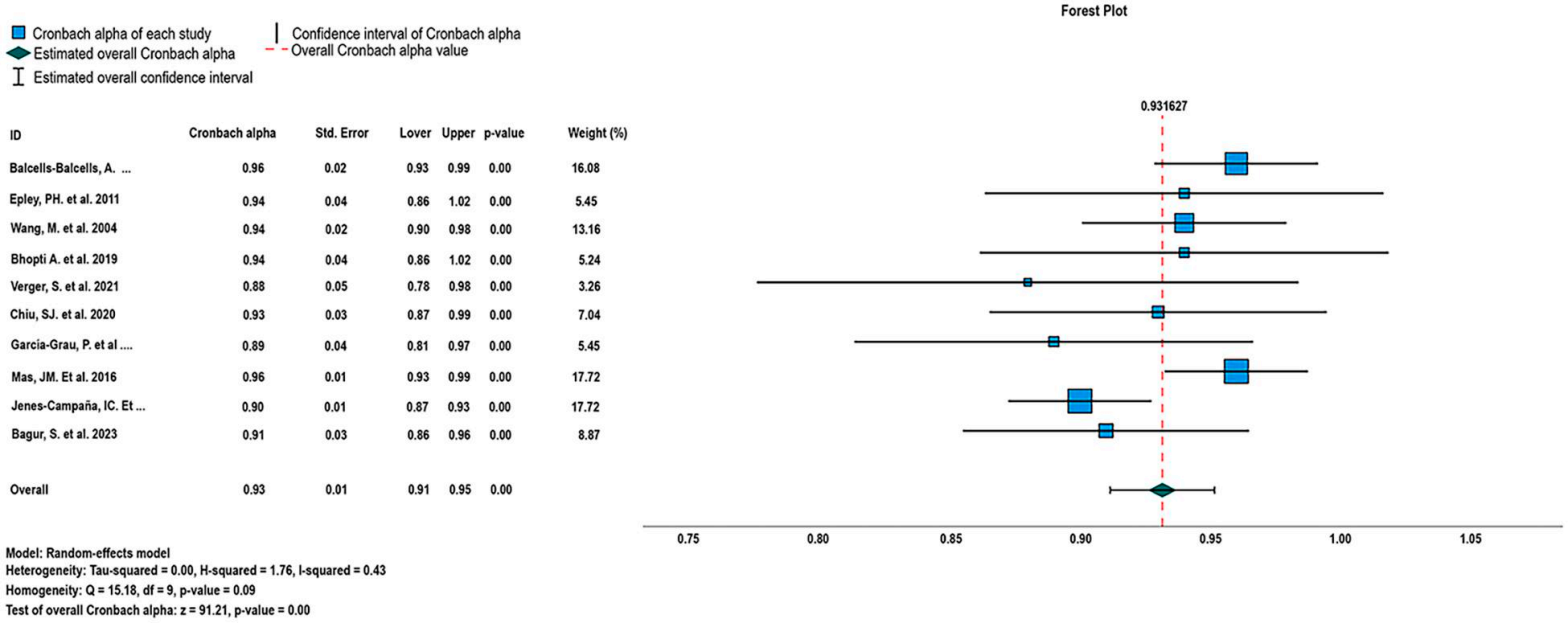
Cronbach's *α* forest plot.

With regard to the weight of the articles, the ones that contribute the most to the study are those of Mas et al. (21) and Jemes-Campaña et al., with 17.7%, followed by Bacells-Bacells et al., with 16.1%. The articles that least contribute to the study are those of Verger et al., with 3.3%, followed by Bhopti et al., with 5.2% (Figure 2).

#### 3.4.2 FQOL

The pooled FQOL mean was 3.812 (95% CI: 3.464–4.160) was obtained, standard error (0.1776) (Table 3). The homogeneity test obtained a significance level of 0.874 (Table 4). In the global FQOL test, the *p* value was below 0.01.

**Figure 3:**
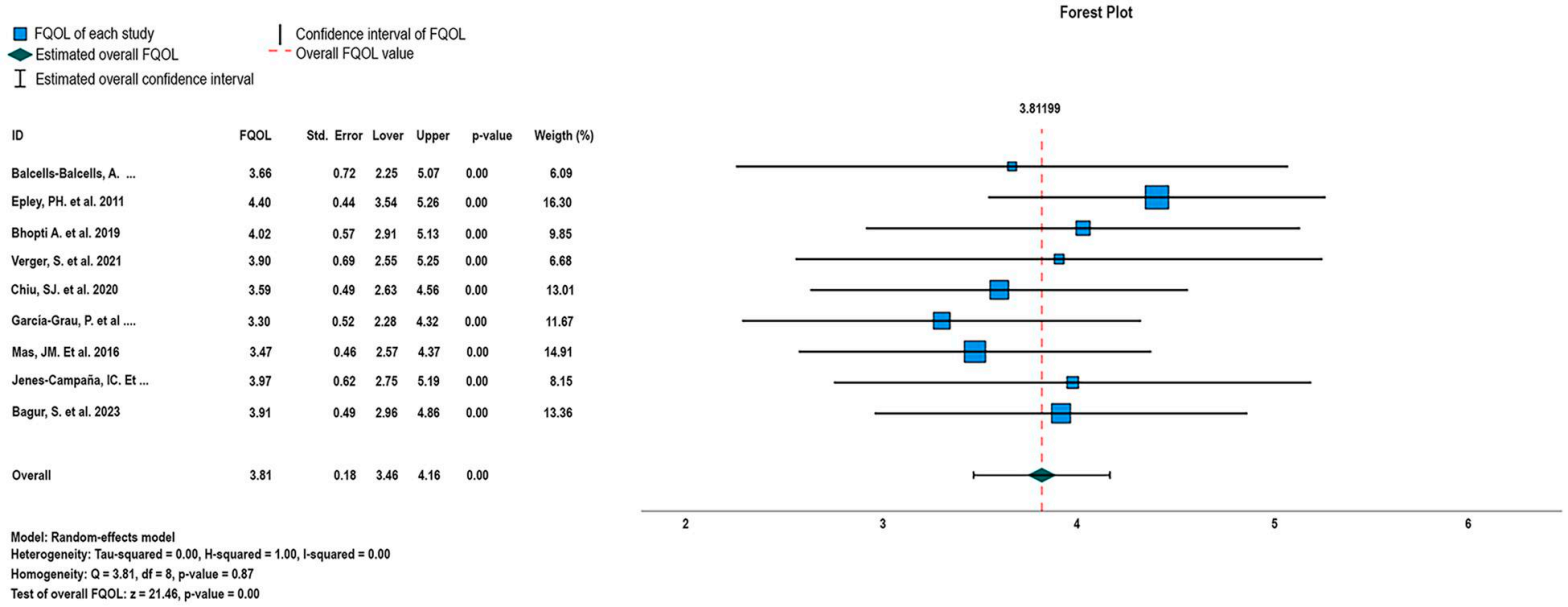
FQOL forest plot graph.

The article with the greatest weight was that of Epley et al., with 16.3%, followed by that of Mas et al. (21), with 14.9%, whereas the article with the lowest weight was that of Balcells-Balcells. et al., with 6.1%, followed by that of Jemes-Campaña et al., with 8.1% (Figure 3).

## 4 Discussion

The objectives of this systematic review and meta-analysis were to examine how FQOL is conceptualized in this context and identify the most frequently assessed domains and instruments used; identify and describe the key factors reported in the literature that may influence family quality of life in EI contexts, based on patterns observed in quantitative findings and narrative synthesis. Therefore, we discuss the results analyzed based on each of the dimensions and variables related to FQOL.

### 4.1 Family quality of life

The results of the articles included in the review may indicate that the levels of FQOL of families with a child that attends EI are high (8, 12–14, 18, 21, 22), which is in line with another study (24) and can be explained through different hypotheses.

Bacells-Bacells et al. stated that the work conducted in EI centers and the fact that intervention is being provided increasingly earlier may have positive effects on FQOL; moreover, FQOL is especially influenced by how professionals provide these supports to the children and parents, showing positive association (13). Furthermore, the study of Epley et al. shows that the extent to which EI services satisfy the families influences their FQOL level (14). This is in agreement with the longitudinal study of Chiu et al., where FQOL decreased with the termination of the intervention due to a lack of resources, thus they asserted that the improvement of FQOL experienced by the families when they begin the intervention may be due to family support and access to resources (8).

Mas et al. explained that the scores of the scales that measure FQOL tend to be higher than those that assess results based on negative aspects such as depression, stress, caregiver burden and pessimism (21), which is corroborated by a previous study, which proposes the FQOL scale for being more neutral, although it also enables the expression of the negative emotional impacts on the “emotional well-being” subscale (25). On the other hand, the qualitative results of Bhopti et al. assume that the FQOL results were related to the inherent attributes of the families, mainly their positive attitudes, their beliefs about their parental duties, and their system of values, as well as their hopes on the successful progress of their children in the future (22).

With respect to the statistical analyses, Cronbach's *α* was included in the present review as an indicator of the internal consistency of the FQOL measurement instruments reported across studies. Although *α* does not offer a comprehensive psychometric assessment, it remains one of the most consistently reported indices of reliability in observational studies and provides insight into how well the items within each instrument perform as a unified measure (26). The global Cronbach's *α* value was potentially high, and the test reached statistical significance, suggesting that the FQOL instruments used in the included studies generally could demonstrated acceptable internal consistency.

Similarly, the global effect size for FQOL, based on standardized scores, appeared relatively high, which may indicate that families of children attending Early Intervention services tend to report favorable levels of family quality of life. The clinical interpretation of the pooled FQOL scores was based on standardized ranges derived from the 1–5 Likert scale used across studies. Following a common approach for interpreting Likert-type scales, the range was divided into three equal intervals: low (1.00–2.33), moderate (2.34–3.66), and high (3.67–5.00) (27). Based on this classification, the pooled mean FQOL score of 3.812 can be interpreted as falling within the high range, although close to the upper limit of the moderate category. This suggests that families receiving Early Intervention services generally report relatively favorable levels of family quality of life. Although the results obtained in the homogeneity test were not significant, there was some disparity among the articles, which could be due to the heterogeneity among the articles in the population included, in terms of both the diagnosis of the child population and the degree of disability.

### 4.2 Support needs of the families

The studies included in this review suggest that the supports provided to the children and their families have a positive effect on their FQOL (8, 13). The articles point out that EI services satisfy their needs (14). EI could help the families to identify their strengths, which has positive outcomes in family resilience and, therefore, in FQOL (1, 8). The families of children who receive EI show a medium-high empowerment level, and the higher the level of empowerment, the greater the FQOL level, and vice versa (18).

Among the most solicited supports by the families was the information provided by the professionals about the state of their children (22) and about the alert signs in the development of the latter to allow them to solicit supports (13). This is coherent with the study of Summers et al., where 73 families stated that they needed information about where they could obtain services for their children, although only 17 of them received this type of service (25). In the study of Escorcia-Mora et al., the families valued the information provided; however, verbal transmission did not imply that the families understood and acquired enough strategies to incorporate the new guidelines to their daily routines, thus feedback modeling and coaching techniques must be included in the interventions (28). This is also corroborated by another study (29), which asserts that information empowers and helps the families to understand the capacities and needs of their children, allowing them to interact with the latter in a way that their development and skills are maximized.

### 4.3 Family characteristics

Family income could be one of the most predictors of FQOL. Higher socioeconomic status was associated with better FQOL outcomes, likely due to increased access to resources and reduced financial stress (12, 18, 19, 21, 22). FQOL is conditioned by yearly income, which has an impact on family climate, emotional stability, economic well-being, adaptation, family resources and even parental physical health, as they reported that they had suspended their own healthcare needs due to economic restrictions and the needs of their children (18, 22). On the contrary, Chiu et al. did not find a significant association between income and FQOL, reporting that the population of their study had medical insurance (8). This is consistent with a previous review and other studies in which the results determine that family resources, such as income, influence FQOL, as they allow them to face any adversities that emerge with regard to the disabilities of their children (30–32).

The results of the studies show a negative and significant association for the severity of the disability and the satisfaction of the families with FQOL (19, 21). The type of disability also could influenced FQOL, according to García-Grau et al., with the families of ASD children showing the lowest scores (12). This could be explained by the alteration in communication and behavior, which has an impact on parental stress (12). This is in line with the findings of another study, which reports that the families of children with behavioral alteration experienced lower levels of FQOL (31). Moreover, having or not having a diagnosis influences the family behavior and, to a greater extent, the family climate, since parents need informative support in order to understand the condition of their children (18). However, the studies of Epley et al. did not find a relationship between the type of disability and FQOL (14); the same was observed in Davis & Gavidia-Payne, who justified this based on the fact that 84.3% of the participants of their study were in the mild- and moderate-level groups, whereas only 15.6% were classified as severe (31).

The families that received support from a single professional, and thereby obtained a transdisciplinary intervention, showed lower levels of FQOL than those who attended more services (8, 12), which could be due to the saturation of the families for attending different institutions (8). The families that received a family-centered intervention model obtained higher scores (12); however, the type of intervention did not have an impact on FQOL in the study of Bagur et al. (18). In addition to these results, the pre- and post-intervention study of Frugone-Jaramillo & Gràcia indicates that family-centered intervention creates opportunities that improve, support and strengthen family functioning, thus improving FQOL (33).

### 4.4 Satisfaction with the service

Thae results suggests that families in general are satisfied with the intervention received (7). The perceived quality of the service was associated positively with both FQOL and satisfaction; moreover, satisfaction was associated with FQOL (20). These results are supported by a systematic review (34), which states that, due to this relationship, there is an improvement in the competencies related to the quality of the service.

Regarding the most valued aspects, there is a positive relationship between the family and the professional (7), and families consider that a relationship of trust and balance is essential (13). This is supported by the results of a different study, in which one of the items best valued by the families was the relationship with the professional (25). The valuations of the families differ in best valued aspects as a function of the type of intervention they perceived, in a way that the families that received home intervention were satisfied with the process of adaptation of the professional to the upbringing style and family dynamics, and the families that received center intervention valued the perspective of the specialized professional, as well as the information received (7).

On the contrary, the aspects that were worst valued by the families were the period of evaluation prior to admission to the EI program, given the waiting lists and delayed entry (7), and the lack of services and support during the transition to the school system, since they feared losing the economic, emotional and communication support from the EI center and its professionals (22). According to another study conducted by Summers et al., the families were more satisfied with the professionals who worked with their children and the families themselves than with aspects related to the service system, such as the number of interventions, which is consistent with the results of this review (25).

It is also important to acknowledge several limitations of this review. First, the lack of standardization in the assessment tools used across studies. Although the Beach Center Family Quality of Life Scale was the most frequently employed instrument, four different scales were identified overall. This variability may have introduced inconsistencies in the measurement of family quality of life and complicated the direct comparison and synthesis of results across studies. Since FQOL is a recent concept in research (35, 36), there are few articles that measure FQOL in families that attend EI, thus further studies with larger samples are needed to measure the FQOL of this population.

A major limitation is that most of the included studies were rated as having medium methodological quality. Specifically, 8 out of the 10 included studies achieved only moderate quality scores, which may limit the robustness and generalizability of the pooled findings. These findings highlight the urgent need for future high-quality research in this field, including studies with stronger methodological designs, larger and more representative samples, standardized outcome measures, and more rigorous reporting procedures.

Another limitation of this review is the heterogeneity of the samples across the included studies, particularly regarding the types of developmental disorders and the risk of developmental delay. This variability may have influenced the results presented in Figure 3, introducing some degree of inconsistency in the reported levels of FQOL. However, this heterogeneity also accurately reflects the real-world population served by EI programs, which includes a wide range of diagnoses and developmental risk conditions.

Finally, the inclusion of studies published only in English may have introduced language bias. As a result, relevant studies published in other languages may have been excluded, potentially limiting the comprehensiveness and generalizability of the findings.

## 5 Conclusions

Statistical analyses Statistical analyses indicated that the FQOL instruments used across studies generally showed acceptable internal consistency, while FQOL scores were relatively high in families of children receiving Early Intervention. This positive outcome may stem from the beneficial impact of EI on family dynamics, influenced by several factors. A strong professional-family relationship effectively meets families' support needs, while the intervention helps families identify their strengths, thereby enhancing family resilience. Additionally, reported satisfaction with services, which was favorable across studies, positively affects FQOL.

The review identified various instruments used to measure FQOL, with the Beach Center Family Quality of Life Scale (BC-FQOLS) being the most frequently employed and exhibiting high internal consistency, supporting its reliability in this population.

Furthermore, several factors were consistently associated with variations in FQOL levels, including family income, type and severity of disability, presence of diagnosis, professional support, and satisfaction with the intervention. Family-centered and transdisciplinary approaches, empowerment, and access to information were also positively linked to FQOL outcomes.

In summary, Family Quality of Life in the context of Early Intervention is generally high but influenced by both family-specific and service-related variables. These findings underscore the necessity for EI programs to adopt comprehensive, family-centered practices that extend beyond child-focused interventions to address the broader needs of families. Further research is warranted to standardize measurement tools and explore underrepresented domains of FQOL.

## Data Availability

The original contributions presented in the study are included in the article/Supplementary Material, further inquiries can be directed to the corresponding author/s.
