# Supplementary material for: Family quality of life in early intervention: systematic review and meta-analysis
Source: Front Pediatr. 2026 Jun 24;14:1866502. doi: 10.3389/fped.2026.1866502 (PMC13341608; doi:10.3389/fped.2026.1866502)
Supplement: Supplementary file 1 [file Supplementaryfile1.docx]

| Database | Search Strategy | Fields Searched | Filters Applied |
| --- | --- | --- | --- |
| PubMed | (“developmental disabilities” OR “developmental disorders” OR “developmental delay” OR “global delay”) AND (“early intervention” OR “early childhood intervention”) AND (“quality of life” OR “family quality of life”) | Title/Abstract | None |
| Scopus | (“developmental disabilities” OR “developmental disorders” OR “developmental delay” OR “global delay”) AND (“early intervention” OR “early childhood intervention”) AND (“quality of life” OR “family quality of life”) | Title/Abstract/Keywords | None |
| CINAHL | (“developmental disabilities” OR “developmental disorders” OR “developmental delay” OR “global delay”) AND (“early intervention” OR “early childhood intervention”) AND (“quality of life” OR “family quality of life”) | Abstract (Advanced Search) | None |
| Web of Science | (“developmental disabilities” OR “developmental disorders” OR “developmental delay” OR “global delay”) AND (“early intervention” OR “early childhood intervention”) AND (“quality of life” OR “family quality of life”) | Abstract | MESH headings: *child/family relations* OR *family* OR *infant*; MESH Qualifiers: *rehabilitation* OR *education* OR *psychology* |

**APPENDIX 1**
